# The effects of transcranial magnetic stimulation on cognitive flexibility among undergraduates with insomnia symptoms: A prospective, single-blind, randomized control trial

**DOI:** 10.1016/j.ijchp.2025.100567

**Published:** 2025-04-14

**Authors:** Muyu Chen, Jun Jiang, Han Chen, Xinyu Liu, Xinpeng Zhang, Li Peng

**Affiliations:** a Department of Military Psychology, School of Psychology, Army Medical University, Chongqing, China; b Department of Basic Psychology, School of Psychology, Army Medical University, Chongqing, China; c Department of Rehabilitation, Southwest Hospital, Army Medical University, Chongqing, China

**Keywords:** Insomnia, Transcranial magnetic stimulations, Cognitive flexibility, Event-related potential

## Abstract

**Backgrounds:**

Repetitive transcranial magnetic stimulation(rTMS) has been widely used in the treatment of insomnia, but there is a lack of research on whether this method could enhance the cognitive flexibility(CF) of individuals with insomnia symptoms.

**Objectives:**

To investigate the effects of rTMS on the CF of undergraduates with insomnia symptoms.

**Methods:**

29 participants were randomly assigned into Active group(*n* = 15) and Sham group(*n* = 14), receiving 1 Hz rTMS interventions targeting the left dorsolateral prefrontal cortex for 2 weeks, comprising 10 sessions (active vs sham stimulation). Sleep quality and CF were assessed using the Pittsburgh Sleep Quality Index(PSQI), Insomnia Severity Index(ISI), Cognitive Flexibility Inventory(CFI), and the Number-Letter Task (N-L task) at baseline(T0), post-intervention(T1), and 8 weeks’ follow-up(T2), and event-related potential(ERP) data during the N-L task were recorded.

**Results:**

Following the intervention, compared to the Sham group, the ISI and PSQI scores in the Active group were significantly decreased, and the CFI score was significantly increased (*P* < 0.01); the results of the N-L task indicated that at T1, the switch cost of reaction time and accuracy for the Sham group were significantly higher than those for the Active group(*P* < 0.05). ERP analysis indicated that at T2, under switch conditions, the amplitude of the frontal area P2 in the Active group was significantly greater than that in the Sham group, and the beta-band ERD at parietal region in the Active group was significantly lower than that in the Sham group (*P* < 0.05).

**Conclusions:**

rTMS could improve sleep quality and enhance the CF of undergraduates with insomnia symptoms.

**Clinical Trials Registration:**

The effect of transcranial magnetic stimulation on cognitive flexibility in college students with insomnia (ChiCTR2400081263) URL: https://www.chictr.org.cn/showproj.html?proj=202951

## Introduction

### Insomnia and cognitive flexibility

Insomnia is characterized by a subjective experience where individuals, despite having adequate opportunities for sleep and a favorable sleep environment, are still dissatisfied with the duration and (or) quality of their sleep, and this dissatisfaction impairs their daytime functioning or leads to physical discomfort(World Health Organization, 2021). Approximately 19∼50 % of adults have been affected by insomnia (Sutton, 2021), and the prevalence of insomnia among undergraduates is not negligible. The detection rate of insomnia among undergraduates in western countries ranges from 18.1 % to 29.7 % (King et al., 2023), while the detection rate of sleep problems among Chinese undergraduates is as high as 23.5 % (Y. Chen et al., 2022). Insomnia exerts a range of adverse effects on the physical and psychological development of undergraduates, with its influence on cognitive flexibility being particularly notable. As one of the core components of executive functions, cognitive flexibility refers to the advanced cognitive ability that allows individuals to adapt by switching between behaviors, perspectives, and strategies in a dynamic environment to take appropriate actions(Diamond, 2013). Cognitive flexibility begins to emerge in early childhood and continues to develop as the prefrontal cortex (PFC) and other regions mature, remaining plasticity even into early adulthood(Dajani & Uddin, 2015; Ganesan & Steinbeis, 2022). Brain functional imaging studies reveal that the brain functional network basis of cognitive flexibility mainly includes the lateral frontal-parietal network (FPN), the default mode network (DMN), and the mid-cingulo-insular network (M-CIN)(Friedman & Robbins, 2022). Particularly, the PFC and the FPN are the most critical neural substrates for cognitive flexibility: Yuan and Raz (2014) indicated that the volume and thickness of the PFC were positively correlated with cognitive flexibility, and the study of Yao et al. (2020) suggested that the FPN might be involved in the process of set shifting within cognitive flexibility. Numerous studies have proved the detrimental impact of insomnia on cognitive flexibility. For instance, a study focusing on Chinese children aged 6–8 years demonstrated that children who reported shorter sleep durations exhibited poorer performance on the Wisconsin Card Sorting Test, a widely used measure of cognitive flexibility(Chen et al., 2021). Research by Ballesio et al. (2020) indicated that insomnia symptoms play a moderating role in the relationship between perseverative cognition and performance on task-switching paradigms. Furthermore, Batzikosta et al. (2024) found that among individuals with mild cognitive impairment, the severity of insomnia was a significant predictor of cognitive flexibility performance. A meta-analysis including 22 studies indicated that in the elderly population, insomnia affected various cognitive functions especially for the cognitive flexibility(Kong et al., 2023). Given these findings, investigating the impact of insomnia on cognitive flexibility and developing appropriate intervention measures are of great significance for maintaining individual physical and mental health.

Although the mechanism by which insomnia impacts cognitive flexibility has not been fully elucidated, the existing research results suggest that this influence may be associated with brain networks centered around the PFC: M. Chen et al. (2024) observed significant neurophysiological differences between undergraduates with insomnia and those with normal sleep when engaging in task-switching paradigms. Specifically, it was found that insomnia undergraduates exhibited altered average amplitudes of P2, N2, and P3 components in frontal and parietal regions, as well as differences in event-related spectral perturbations within theta and alpha bands, and Zhang et al. (2024) reported similar findings in adolescents following a 24-hour sleep deprivation period. Kim et al. (2022) found through their study of functional magnetic resonance imaging (fMRI) results of workers with permanent shift work that, compared to the healthy controls, shift workers with long-term disrupted sleep rhythms showed compensatory hyperactivation in the left dorsolateral prefrontal cortex (dlPFC) when processing negative words. An fMRI study involving 11,057 children aged 9–11 years revealed that the volume of the frontal superior gyrus and the frontal middle gyrus was positively associated with sleep duration and cognitive performance(Anastasiades et al., 2022). Brooks et al. (2022) discovered in their resting-state MRI study involving 5566 adolescents that the FPN and the DMN are the most profoundly impacted brain networks by sleep quality degradation, which is marked by shorter sleep duration, longer sleep onset latency, and symptoms of sleep-disordered breathing. Cheng et al. (2018) discovered that increased functional connectivity in regions such as the dlPFC, cingulate cortex, and insula were significantly linked to poor sleep quality and depressive mood, based on their study of brain functional connectivity in 1017 adult volunteers aged 18–59. A meta-analysis by Guarana et al. (2021) indicated that the effect of insomnia on EFs might be realized through reduced activity in the dlPFC and alterations in the functional connections of the entire FPN. Therefore, the low cognitive flexibility in individuals with insomnia may be due to structural or functional changes in the PFC caused by insomnia, indicating that the PFC may be an important target for improving cognitive flexibility in individuals with insomnia.

### Effects of rTMS on insomnia and cognitive flexibility

Intervention methods for insomnia include cognitive behavioral therapy, medication treatment, physical therapy and so on (Perlis et al., 2022). In recent years, as one of the non-invasive brain stimulations (NIBS) technique that employs electromagnetic induction to generate currents to temporarily alter brain cortex activity, repetitive transcranial magnetic stimulation (rTMS) has increasingly drawn the attention of researchers for its potential in treating insomnia (Lefaucheur, 2019). It is generally believed that low-frequency (≤5 Hz) rTMS (LF-rTMS) could reduce cortical excitability by affecting the level of γ-aminobutyric acid (GABA) within the cortex (Lanza et al., 2018), and is therefore often used for treating insomnia(Sun et al., 2021). In 2023, meta-analysis results from Krone et al. (2023). and Lanza et al. (2023) both indicated that rTMS targeting the dlPFC could help alleviate insomnia symptoms. Therefore, in the recently published *“Chinese guideline for diagnosis and treatment of insomnia (2023)”* in June 2024, TMS with a frequency of ≤1 Hz was recommended (Level B) as a monotherapy or adjunctive therapy for alleviating insomnia symptoms(Wang, 2024); and *“The European Insomnia Guideline (2023)”* also encouraged conducting more randomized controlled trials (RCTs) to provide more empirical evidence for rTMS intervention in insomnia (Riemann et al., 2023).

On the other hand, the impact of rTMS on cognitive flexibility has also garnered interest from researchers. As the most common targeted region in rTMS, dlPFC is also a primary neural substrate for cognitive flexibility(Pitcher et al., 2021); in other words, the stimulation position of rTMS coincides with the brain structures involved in cognitive flexibility. Previous studies have indicated that rTMS has a positive impact on cognitive flexibility. A meta-analysis of 24 high-frequency rTMS (HF-rTMS) studies conducted by Xu et al. (2023) pointed out that, after the intervention, subjects showed improvements in various domains of EFs, including cognitive flexibility. Another meta-analysis of 17 rTMS studies suggested that after receiving rTMS intervention, patients with depression experienced improvements in cognitive functions such as working memory and cognitive flexibility (Hernandez-Sauret et al., 2024). Nevertheless, current research on the influence of rTMS on cognitive flexibility is relatively limited, with the study samples predominantly consisting of individuals with depression, obsessive-compulsive disorder, and other psychiatric conditions, and there has been a lack of exploration into its effects on the cognitive flexibility of insomnia individuals.

### Study objectives

Hence, this prospective, single-blind, randomized controlled study aims to explore the impact of rTMS on the cognitive flexibility of undergraduates with insomnia symptoms, and to preliminarily explore its neuro-electrophysiological mechanisms. We propose the following hypotheses:

Hypothesis 1: rTMS could improve the sleep quality of undergraduates with insomnia symptoms and enhances their cognitive flexibility;
Hypothesis 2: The enhancement of cognitive flexibility in undergraduates with insomnia symptoms by rTMS may be linked to certain changes in brain neurophysiological activity.

## Material and methods

### Participants

The recruitment of participants for this study was conducted from August 2023 to June 2024, and all the participants in this study were recruited as volunteers. This research adhered strictly to the principles outlined in the Declaration of Helsinki and had received ethical approval from the Medical Ethics Committee of Army Medical University (approval number: 2023–5–02). It had also been registered with the Chinese Clinical Trial Registry (registration number: ChiCTR2400081263).

#### Sample size

With an effect size of 0.3, *α*=0.05, Power=0.80, group=2, Number of measurements=3, we used the G-power software (v 3.1) to calculate that a total of 28 participants should be recruited; considering an estimated dropout rate of 15 %, therefore, 33 participants need to be recruited for the study.

#### Inclusion and exclusion criteria

Inclusion criteria: (1) Full-time undergraduate students (have passed the National College Entrance Examination of China) with insomnia complains; (2) In good health recently with normal circadian rhythm, able to provide a medical examination report from the past year, which showing there were no physical or mental conditions that could impact sleep; (3) Have not taken any medications that could affect sleep in the last three months; (4) Insomnia Severity Index (ISI) score ≥15 and Pittsburgh Sleep Quality Index (PSQI) ≥8, and meet the diagnostic criteria for primary insomnia as per the International Classification of Sleep Disorders (Third Edition) (ICSD-III)(American Academy of Sleep Medicine, 2014); (5) Have not participated in any psychological interventions or NIBS programs before; (6) Voluntarily participate in this trial and sign a written informed consent form.

Exclusion criteria: (1) Non-undergraduate student or have graduated; (2) Age below 18 years or above 24 years; (3) Having experienced any other sleep disorders (e.g., sleep apnea, restless leg syndrome) or health issues (e.g., surgery for injuries, depression, schizophrenia, depressive disorder, anxiety disorder, bipolar disorder) in the past 6 months that could impact sleep, or having taken substances that may affect sleep (such as melatonin, benzodiazepines, zopiclone, etc.) in the last 6 months; (4) Having an artificial pacemaker or other implanted devices, or metal foreign bodies in the brain; (5) Have suffered from neurological diseases such as stroke or epilepsy, or has a family history of such diseases; (6) Members of disadvantaged groups such as active military personnel, people with disabilities.

Subjects were interviewed by the researchers according to ICSD-III before enrollment to clarify their current sleep status and mental-physical condition, after which they were asked to sign an informed consent form and be included in the study.

#### Questionnaires

We used these following questionnaires to recruit and screen eligible volunteers of our study:

(1) Demographic information: age, sex, grade, recent medical history, height, weight, smoking, and household registration status.
(2) Cognitive Flexibility Inventory (CFI)(Dennis & Wal, 2010): Consisting of 20 items, it uses a Likert scale from 1 to 7 for scoring, divided into two dimensions: selectivity and controllability. A higher score indicates better cognitive flexibility in an individual. In this study, the Cronbach's alpha coefficient for this scale was 0.92.
(3) ISI (Bastien et al., 2001): This index comprises 7 items scored on a 5-point Likert scale ranging from 0 to 4. The scoring criteria are categorized as follows: 0–7 points suggest no insomnia, 8–14 points indicate mild insomnia, 15–21 points suggest moderate insomnia, and 21–28 points indicate severe insomnia. Morin et al. (2011)reported the diagnostic sensitivity and specificity of this index as 99.4 % and 91.8 %, respectively. In this study, the Cronbach's alpha coefficient for this scale was 0.96.
(4) PSQI (Buysse et al., 1989): Comprising 9 items with 18 questions, it includes 7 dimensions: subjective sleep quality, sleep latency, sleep duration, habitual sleep efficiency, sleep disturbances, use of sleep medications, and daytime dysfunction. In the English version of PSQI, a score of > 4 is used as the cutoff, while in the Chinese version, a score of > 7 is used as the cutoff (Lu et al., 2014); Liu et al. (1996) measured the diagnostic sensitivity and specificity of the Chinese version of the PSQI as 0.98 and 0.90, respectively. In this study, the Cronbach's alpha coefficient of choice questions of this scale was 0.86.

The questionnaires, which were integrated into an electronic format, were disseminated through the Wenjuanxing online platform (www.wjx.cn) and during various educational activities, such as extracurricular classes. The administration of the questionnaires was conducted in accordance with the principles of voluntary participation and confidentiality. Participants were mandated to acknowledge the informed consent before filling out the questionnaire, with automatic exit from the survey if this acknowledgment was not provided. They had the right to withdraw from the study at any stage.

#### Grouping

The participants were randomly allocated into an active intervention group (Active group) and a sham stimulation group (Sham group) using a random number table method. The randomization process was as follows: Two researchers each generated a random integer on their computers, and then these numbers were added together. If the sum was odd, the participant would be assigned to the Active group; if the sum was even, the participant would be assigned to the Sham group. The results of randomization were blinded to participants.

**Fig. 1:**
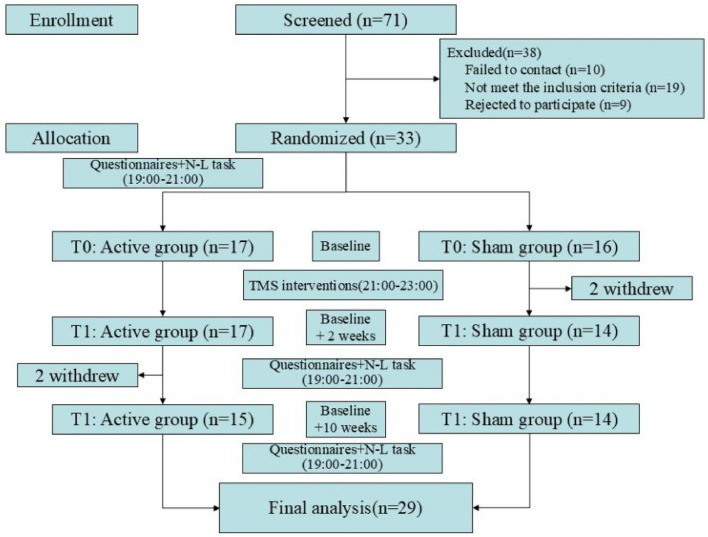
CONSORT flow chart.

A total of 71 volunteers were recruited, of which 10 were failed to contact, 19 did not meet the inclusion criteria, and 9 withdrew due to their inability to participate in the entire study. Therefore, a total of 33 participants were included in this study, with 17 in the Active group and 16 in the Sham group. During the study, 2 participants (Sham group: 2) dropped out due to dissatisfaction with the intervention effects; and 2 participants (Active group: 2) failed to complete the follow-up. Consequently, a total of 29 participants were included in the final analysis, with further details provided in Fig. 1. There were no significant differences in sociodemographic information between the two groups of participants, details can be found in Table 1.

**Table 1:** Comparisons of sociodemographic information ($\bar{x}\pm s$).

Variables	Active group (*n* = 15)	Sham group (*n* = 14)	$t/\chi^{2}$
Age(yrs)	20.21±0.89	19.80±0.68	1.42
Grade (2/3)	7/8	6/8	0.04
Sex(male/female)	5/10	4/10	0.07
BMI (kg/m^2^)	21.21±2.35	20.92±2.14	0.34
Smoking (yes/no)	3/12	2/12	0.16
Household (city/country)	9/6	7/7	0.29

Abbreviations: BMI: body mass index;.

### rTMS intervention

In this study, all rTMS interventions were performed using the Magpro-X100(Magventure, Farnum, Denmark) system; and the localization of various brain regions was accomplished using the international 10–20 system. Participants wore nylon caps (Yingzhi, Shenzhen, China) that corresponded to the 10–20 system, and the localization process was completed according to the markings on the caps. Before interventions, the resting motor threshold (RMT) of the participants should be determined (Li et al., 2024): Place the recording electrode on the belly of the abductor pollicis muscle and the reference electrode on its tendon, ensuring they are 2–3 cm apart. Position the figure-of-eight coil (C-B60) over the contralateral primary motor cortex area (C3). Gradually increase the stimulation intensity from 20 % of the maximum output strength, increasing by 5 % each time, until the minimum intensity that can evoke a motor evoked potential amplitude greater than 50 μV in at least 5 out of 10 consecutive stimulations of the abductor pollicis muscle is reached. The minimum intensity induced is the participant's RMT.

To prevent scheduling conflicts with participants’ class and maintain intervention quality, all interventions were conducted from 21:00 to 23:00, implemented by two qualified physicians (both of them are capable of independently operating rTMS and performing cardiopulmonary resuscitation, as well as handling emergencies related to epilepsy). During the formal intervention, participants remained seated and were provided with earplugs, earmuffs, and other hearing protection devices if they needed. The parameters for the Active group were as follows: The center of the circular coil (MCF-125) was held tangentially to the l-DLPFC (F3), at an angle of approximately 45° to the skull surface. The magnetic stimulation frequency was set at 1 Hz, and the intensity was 80 % of the RMT. Every 10 s of stimulation was followed by a 2-second pause, with one session per day, totaling 1200 stimulations per session. The intervention lasted for 24 min, and a total of 10 sessions of intervention was conducted 5 days/week for 2 consecutive weeks. Participants in the Sham group received sham rTMS interventions, where the coil was positioned vertically against the subject's skull, thus unable to generate any magnetic field. All other rTMS parameters and treatment schedules were identical to those in the Active group. All adverse events that occurred during the intervention would be strictly documented as a basis for assessing safety. Participants had the right to withdraw the study at any time.

### Behavioral task and ERP recording

The procedures of behavioral trials and ERP acquisition in this study were similar to those in a previous published study (M. Chen et al., 2024). All participants were required to complete the behavioral task at baseline (T0), the day after the intervention completion (T1), and 8 weeks follow-up (T2), while the EEG data were recorded simultaneously when they were performing the task. The experiments were conducted between 19:00 and 21:00 to mitigate the influence of circadian arousal rhythms. The process for recording EEG signals entailed the following steps: Participants were required to wash their hair prior to the experiment, then the EOG electrodes were affixed to the lower right eyelid of the participant using medical pressure-sensitive tape. Next, the participant was fitted with a 64-channel EEG cap produced by Brain Product, Germany (using the ‘FCz’ electrode as the reference) and conductive gel was injected into each electrode on the cap. Once the resistance of all electrodes dropped below 10 kΩ and the EEG signal stabilized, the experiment commenced. The experiments were conducted in a sound-insulated cognitive laboratory, with participants positioned approximately 60 cm from a computer screen. The monitor had a refresh rate of 60 Hz and a resolution of 1920×1080.

**Fig. 2:**
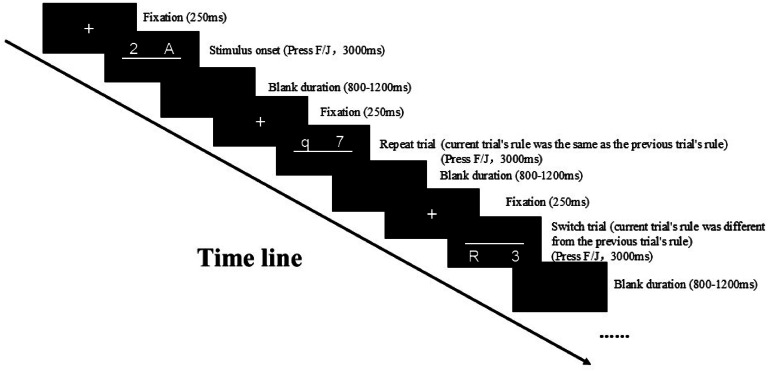
Number-letter task paradigm.

Notes: In this task, participants need to press F/J to make judgments based on the position of the stimulus (above or below the midline): for stimuli above the midline, they were to press the F/J keys to ascertain if the number was greater than 5; for stimuli below the midline, they should determine if the letter was uppercase or lowercase. RT and Acc of different conditions (repeat vs switch) would be recorded, and the switch cost was used for judging the level of cognitive flexibility.

Participants were asked to complete the following behavioral task: employing E-prime 2.0 software, a classic “number letter task” (N-L task) paradigm (derived from the task-switching paradigm) (Schmitter-Edgecombe & Langill, 2006) was utilized to assess the cognitive flexibility of participants. The task comprised 2 practice blocks and 4 formal blocks. Each practice block contained 16 trials, totaling 32 trials, and participants must achieve an accuracy rate of 80 % or higher to proceed to the formal experiment; the formal experiment has 64 trials per block, totaling 256 trials. Stimuli were presented using a pseudo-randomization method: the sequence of stimulus presentation within each block was established in advance through computer-randomized arrangements to counterbalance the number of repeat and switch trials; the order of block presentation was also randomized between different participants. At the beginning of each trial, a “+” fixation appears on the screen for 250 milliseconds (ms); then, the midline and target stimulus are presented, and participants are to press the corresponding key to make a judgment as quickly as possible; the maximum duration of the target stimulus was 3000 ms; after the participant made a response or exceeded the response time limit, an 800–1200 ms blank screen appeared. This task involved presenting a mixed stimulus comprising a letter (either uppercase A/G/Q/R or lowercase a/g/q/r) and a number (1, 2, 3, 4, 6, 7, 8, 9) in the designated stimulus area. Participants were instructed to press the F/J keys to make judgements: for stimuli above the midline, they were to ascertain if the number was greater than 5; for stimuli below the midline, they should determine if the letter was uppercase or lowercase (the process is shown in Fig. 2). For example, when the stimulus “q 7″ appears above the midline, participants need to ignore the existence of “q” and judge whether “7″ is greater than 5; conversely; when “q 7″ appears below the midline, participants need to ignore “7″ and judge whether “q” is uppercase. To test the performance of participants when they need to switch their cognition, this task recorded the reaction time (RT) and accuracy (Acc) in repeat conditions (when the current trial's rule was the same as the previous trial rule, i.e., both the current and previous stimuli appeared on the same side of the midline, a total of 126 trials) and switch conditions (when the current trial's rule is different from the previous trial rule, i.e., the current and previous stimuli appear on different sides of the midline, a total of 126 trials). Switch cost (the difference in RT and Acc between switch trials and repeat trials) was used for judging the level of cognitive flexibility.

### Outcomes

The primary outcomes were CFI, ISI, and PSQI. Secondary outcomes included performance on the N-L task and ERP results.

According to the requirements of the Medical Ethics Committee, to maximize the benefits for the participants, all participants would be informed at T2 that they had completed all tasks of this study. If they needed, they could receive up to 10 sessions of free rTMS (all active stimulations) interventions. In addition, all participants who complete the study would receive a reward of 400 CNY (approximately 50 EUR).

### Data statistics

The statistical analysis of the research results was conducted using the SPSS 26.0 (IBM Corp. Armonk, New York, United States) and MATLAB R2022b (The MathWorks Inc. Natick, Massachusetts, United States) software packages. All statistical tests were performed after removing outliers (i.e., data greater than the mean + 3 standard deviations or less than the mean - 3 standard deviations). Significance was determined at a threshold of α=0.05. In general, for most analyses we reported the original *P*-values; and both unadjusted and Bonferroni-adjusted *P*-values (*P*_adj) were reported when involving multiple comparisons.

#### Basic information, questionnaires, and behavioral data

Questionnaires results and behavioral task data were analyzed using SPSS, and Graphpad prism 8.0 (GraphPad Software, Boston, Massachusetts, United States) was used for visualization purposes. Questionnaires, RT and Acc were examined using a mixed design ANOVA (Time * Group) and tested for main effects and simple effects of the Group (i.e., Active group VS Sham group) and the Time (i.e., T0 VS T1 VS T2) separately. To further reveal the relationship between improvements of insomnia and cognitive flexibility, we conducted additional analyses by performing the Pearson correlation analyses between the change of questionnaire results across different timepoints (δ1: T1-T0; δ2: T2-T0; δ3: T2-T1). Independent samples *t*-tests were conducted to examine differences between groups.

#### ERP data

ERP data were analyzed using the EEGLAB v2023.1 package in MATLAB. The preprocessing parameters were set as follows: (1) Bandpass filtering (range of 0.3∼45 Hz); (2) Pre-segmentation (1000 ms prior to and 2000 ms following stimulus presentation), and removing all error trials and trials with excessively long or short reaction times; (3) Interpolating bad channels; (4) *Re*-referencing (reference electrodes set to bilateral mastoid electrodes ‘TP9’, ‘TP10’); (5) Independent Component Analysis (ICA) and manually removing artifacts; (6) Removing extreme trials (voltage fluctuations exceeding ±100μV). After preprocessing, the EEG data were averaged and calculated for each group of participants under different conditions were plotted using the built-in plotting tool (eegplot).

Time-domain analysis: The time window for ERP time-domain analysis was set from −200 ms before to 800 ms after stimulus presentation, with 200 ms pre-stimulus as the baseline. Based on the waveform observation results and previous literature studies(Romero-Ferreiro et al., 2022; Zhuo et al., 2021), the time windows for analyzing the components of ERP were determined as follows: frontal P2 at T0, T1, T2 and parietal P2 at T2 (160–240 ms), parietal P2 at T0, T1 (200–280 ms), N2 (280∼340 ms), and P3 (340∼420 ms); the analysis regions were as follows: frontal region (F3, F4, Fz), parietal region (P3, P4, Pz). After calculating the average of the data of each electrode in each region, a mixed design ANOVA (Time * Group) was performed for each time-domain component, and the main effects and simple effects of the Group and Time were tested separately.

Time-frequency analysis: The event-related spectral perturbation (ERSP) of the participants was calculated using the short-time Fourier transform (STFT). The time window for ERP time-domain analysis was set from −1000 ms before to 2000 ms after stimulus presentation, with the analysis time step set at 400 ms; the frequency range was 1–30 Hz, and the frequency step was set at 1 Hz; to avoid edge effects, the baseline was set from −800ms∼−200 ms pre stimulus. According to the image observation results and previous literature studies(Bai et al., 2023; Q. Chen et al., 2022; Siqi-Liu et al., 2022), the analysis time window for theta band (4–7 Hz) was set at 100–400 ms, while time window for alpha band (8–13 Hz) was set at 400–1000 ms, and time window for beta band (14–30 Hz) was set at 100–1000 ms. After calculating the average of the data of each electrode in each region, a mixed design ANOVA (Time * Group) was performed for each time-domain component, and the main effects and simple effects of the Group and Time were tested separately.

## Results

### Primary outcomes: questionnaires

No statistically significant differences were found in CFI, ISI, and PSQI between the two groups at T0. The results of the mixed-design ANOVA showed that the interaction effects of CFI, PSQI, and ISI were all significant (*P* < 0.01), and both the main effects of Group and Time of ISI and PSQI were also significant (*P* < 0.001); simple effect analysis indicated that at T1 and T2, PSQI and ISI in the Active group were significantly lower than those in the Sham group (*P* < 0.01); at T1, the CFI in the Active group was significantly higher than that in the Sham group (*P* < 0.05); and at T2, the CFI in the Active group was higher than that in the Sham group, with a marginally significant difference (*P* = 0.07). Pearson partial correlation analysis showed that, after adjusting for potential confounders including age, grade, sex, BMI, smoking status and household registration, compared to T0, the changes in CFI at T1 and T2 were significantly negatively correlated with the changes in ISI and PSQI (*r* > 0.40, *P* < 0.05). Details could be found in Table 2, Fig. 3 and Supplementary Table 1.

**Table 2:** Comparison of Questionnaires ($\bar{x}\pm s$).

Questionnaires	Time	Active group	Sham group	*t*		*F*	
		(*n* = 15)	(*n* = 14)		Interaction	Time	Group
CFI	T0	94.00±12.92	100.29±7.79	−1.60	7.68⁎⁎	1.95	1.58
	T1	107.40±15.13	96.29±10.95	2.25*			
	T2	103.27±17.82	93.50±9.62	1.85			
	Multiple comparison	T0<T1#, T0<T2	-	-			
PSQI	T0	10.13±2.90	10.86±1.96	−0.78	5.50⁎⁎	20.25⁎⁎⁎	15.08⁎⁎⁎
	T1	5.40±2.13	9.00±2.51	−4.17⁎⁎⁎			
	T2	5.60±3.00	9.79±3.02	−3.75⁎⁎			
	Multiple comparison	T0<T1, T0<T2	T0<T1	-			
ISI	T0	16.27±2.92	16.14±1.35	0.15	7.07⁎⁎	31.53⁎⁎⁎	20.22⁎⁎⁎
	T1	6.67±3.76	12.21±4.95	−3.38⁎⁎			
	T2	6.93±4.22	13.29±4.20	−4.06⁎⁎⁎			
	Multiple comparison	T0<T1, T0<T2	T0<T1, T0<T2#	-			

Notes: *: *P* < 0.05.

Abbreviations: CFI: cognitive flexibility inventory; ISI: insomnia severity index; PSQI: Pittsburgh sleep quality index.

⁎⁎ : *P* < 0.01.

⁎⁎⁎ : *P* < 0.001.

# : *P* < 0.05 and *P*_adj>0.05;.

**Fig. 3:**
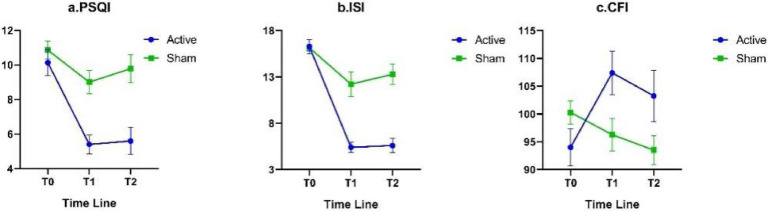
Comparison of Questionnaires.

Notes: CFI: cognitive flexibility inventory; ISI: insomnia severity index; PSQI: Pittsburgh sleep quality index

### Secondary outcomes

#### Behavioral tasks

**Fig. 4:**
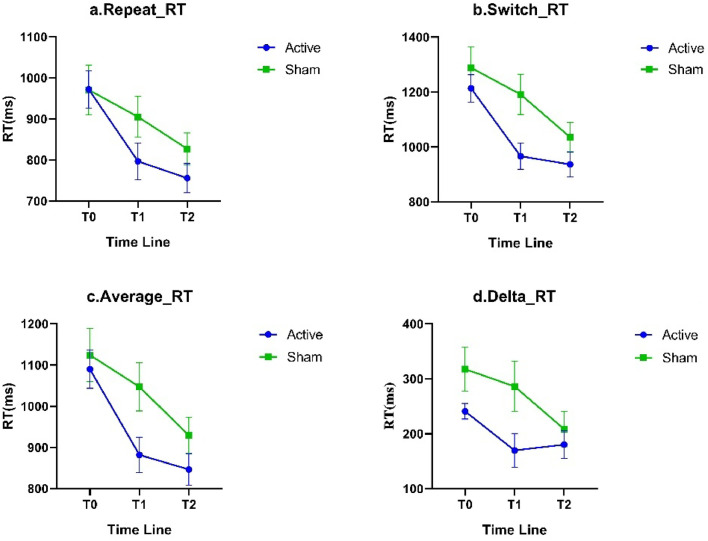
Comparison of RT in N-L task.

Notes: RT: Reaction time; a: Repeat RT; b: Switch RT; c: Average RT; d: Switch cost (RT)

**Fig. 5:**
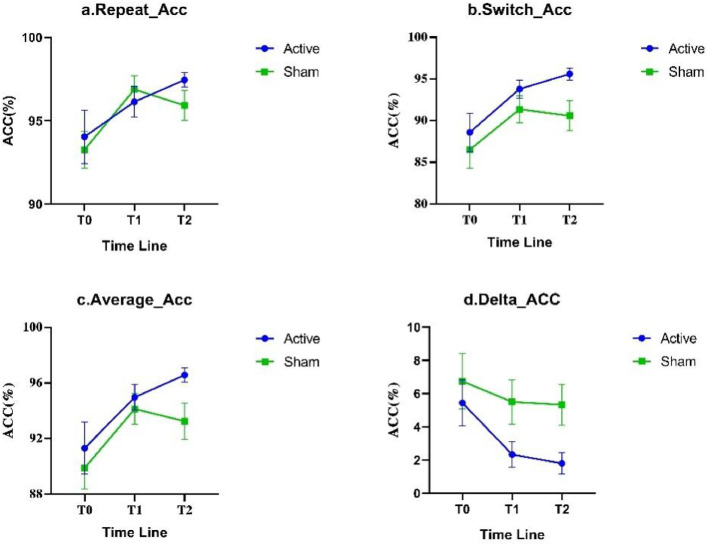
Comparison of Acc in N-L task.

Notes: Acc: accuracy; a: Repeat Acc; b: Switch Acc; c: Average Acc; d: Switch cost (Acc)

At T0, there were no statistically significant differences in all measurements between the two groups; mixed-design ANOVA results indicated a significant interaction effect between switch conditions and average RT (*P* < 0.05); there were significant main effects of Time for all measurement indices (*P* < 0.05); and there was a significant main effect of Group for switch cost of Acc(*P* < 0.05). Simple effect analysis suggested that at T1, the switch cost of RT and Acc in the Active group were significantly smaller than those in the Sham group (*P* < 0.05); at T2, the switch trials Acc and average Acc in the Active group were significantly greater than those in the Sham group, and the switch cost of Acc was significantly smaller than that in the Sham group (*P* < 0.05), see Table 3 and Fig. 4& 5.

**Table 3:** Comparison of behavioral task.

Items	Time	Active group	Sham group	*t*		*F*	
		(*n* = 15)	(*n* = 14)		Interaction	Time	Group
Repeat_RT	T0	972.14 ± 174.37	970.88 ± 225.80	0.02	2.69	29.07⁎⁎⁎	1.02
(ms)	T1	796.86 ± 171.92	905.26 ± 186.15	−1.63			
	T2	755.80 ± 137.43	827.14 ± 144.31	−1.36			
	Multiple comparison	T0>T1, T0>T2	T0>T2, T1>T2	-			
Switch_RT	T0	1213.01 ± 192.94	1288.46 ± 282.93	−0.84	3.64*	40.69⁎⁎⁎	3.22
(ms)	T1	966.41 ± 182.71	1191.48 ± 273.25	−2.59*			
	T2	936.29 ± 173.29	1035.64 ± 198.61	−1.43			
	Multiple comparison	T0>T1, T0>T2	T0>T2^#^, T1>T2	-			
Repeat_Acc	T0	94.04 ± 6.19	93.25 ± 4.10	0.40	1.02	8.83⁎⁎	0.22
( %)	T1	96.14 ± 3.57	96.88 ± 3.01	−0.61			
	T2	97.44 ± 1.68	95.92 ± 3.37	1.53			
	Multiple comparison	T0<T2^#^	T0<T1^#^	-			
Switch_Acc	T0	88.59 ± 8.94	86.51 ± 8.20	0.65	0.70	10.22⁎⁎⁎	2.99
( %)	T1	93.81 ± 4.20	91.38 ± 6.11	1.24			
	T2	95.61 ± 2.78	90.59 ± 6.73	2.59*			
	Multiple comparison	T0<T1, T0<T2	T0<T1^#^	-			
Average_RT	T0	1089.80 ± 180.23	1124.06 ± 241.08	0.59	3.81*	42.82⁎⁎⁎	2.14
(ms)	T1	881.74 ± 166.52	1047.20 ± 219.03	−2.23*			
	T2	846.47 ± 148.12	929.11 ± 163.07	−0.69			
	Multiple comparison	T0>T1, T0>T2	T0>T2, T1>T2	-			
Average_Acc	T0	91.31 ± 7.21	89.88 ± 5.67	−0.02	0.84	11.42⁎⁎	1.82
( %)	T1	94.97 ± 3.60	94.13 ± 4.12	0.58			
	T2	96.58 ± 2.02	93.25 ± 4.81	2.40*			
	Multiple comparison	T0<T1, T0<T2	T0<T1^#^	-			
Switch cost	T0	240.88 ± 54.82	317.58 ± 150.55	−1.80	1.73	6.40⁎⁎	4.04
RT (ms)	T1	169.55 ± 120.06	286.22 ± 169.81	−2.15*			
	T2	180.55 ± 97.66	208.51 ± 120.61	−0.68			
	Multiple comparison	-	T0>T2, T1>T2	-			
Switch cost	T0	5.45 ± 5.36	6.75 ± 6.22	−0.60	0.71	3.72*	4.55*
Acc ( %)	T1	2.33 ± 2.98	5.50 ± 4.99	−2.10*			
	T2	1.80 ± 2.50	5.33 ± 4.55	−2.57*			
	Multiple comparison	T0>T2^#^, T1>T2^#^	-	-			

Notes: *: *P* < 0.05.

⁎⁎ : *P* < 0.01.

⁎⁎⁎ : *P* < 0.001. ^#^: *P* < 0.05 and *P*_adj > 0.05. Abbreviations: RT: Reaction time; Acc: accuracy.

#### ERP

##### Time-domain analysis

**Fig. 6:**
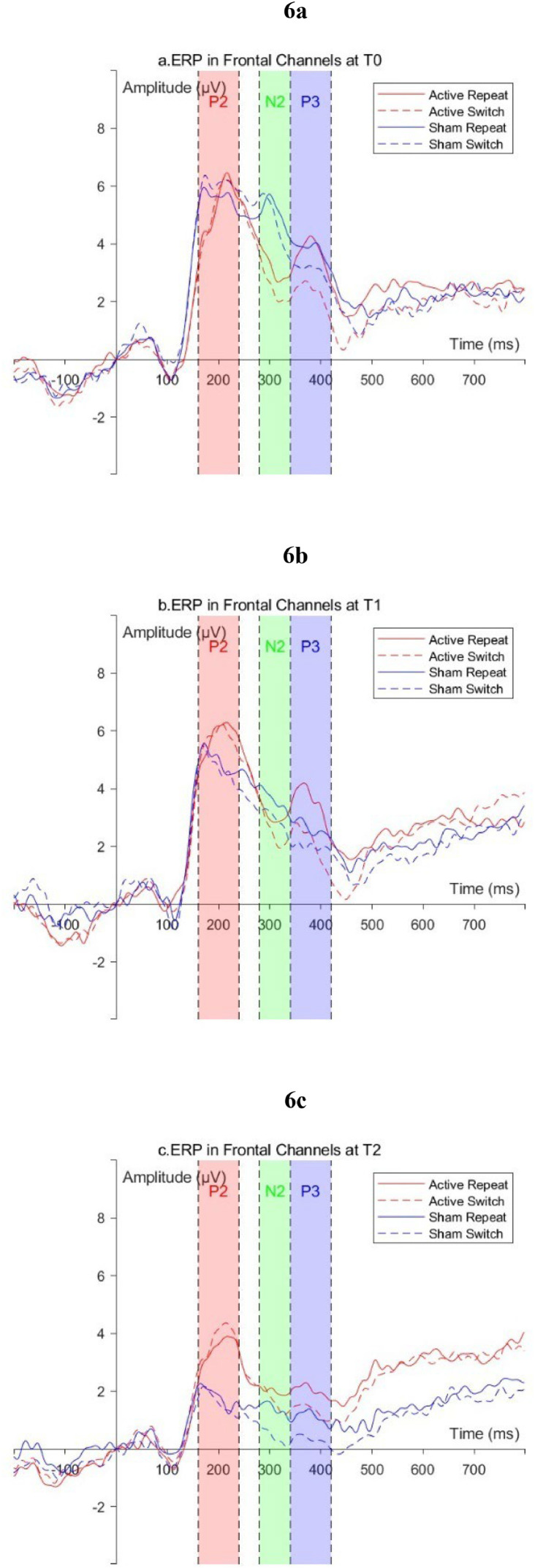
ERP waveforms in the frontal region (F3, F4, Fz) a: T0; b: T1; c: T2.

**Fig. 7:**
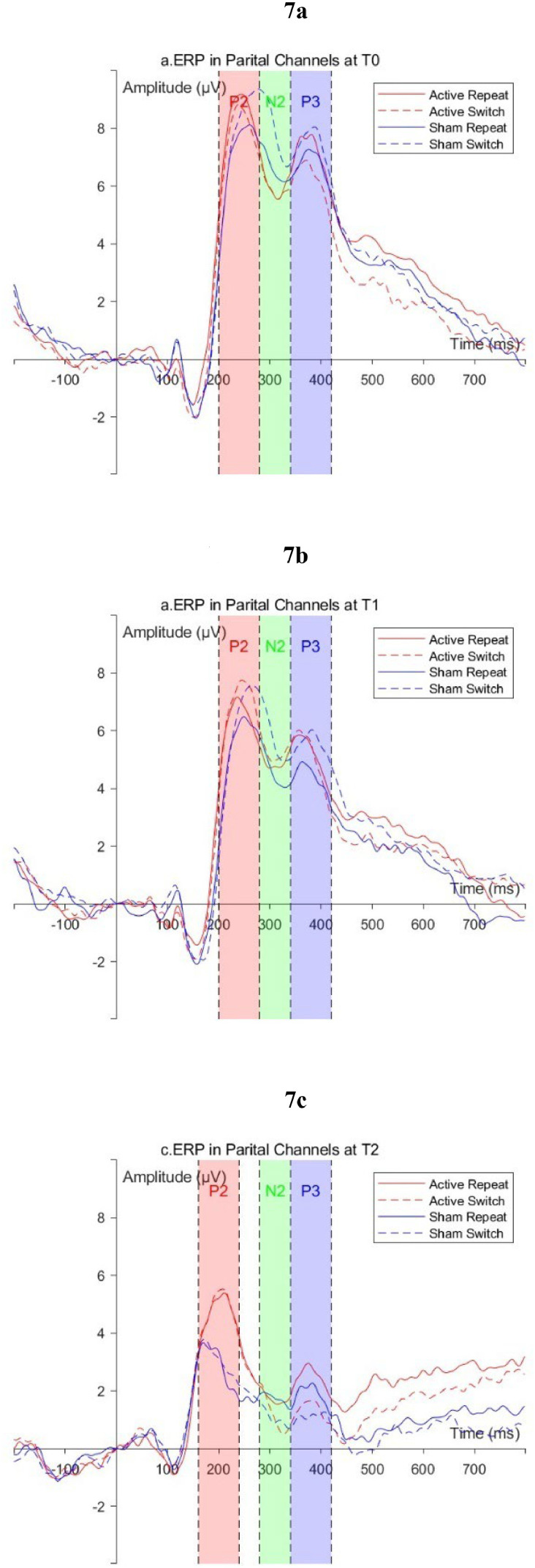
ERP waveforms in the frontal region (P3, P4, Pz) a: T0; b: T1; c: T2.

ERP waveform graphs are shown in Figs. 6–7. The average amplitudes of all ERP components and the comparison results are presented in Supplementary Material Tables 2–4. Mixed-design ANOVA revealed that the main effects of Time for all ERP components in different conditions and different brain regions were all significant (*P* < 0.01); only the interaction effect of the P2 and N2 in the frontal region under switch conditions was significant (*P* < 0.05); simple effect analysis indicated that at T2, under switch conditions, the frontal region P2 amplitude in the Active group was significantly larger than that in the Sham group (*P* < 0.05), and the amplitude in the parietal region was larger than the sham stimulation group, but the difference was marginally significant (*P* = 0.07); under repeat conditions, the frontal and parietal region P2 amplitudes in the Active group were both larger than those in the Sham group, with marginally significant differences (*P* < 0.08).

##### Time-frequency analysis

**Fig. 8:**
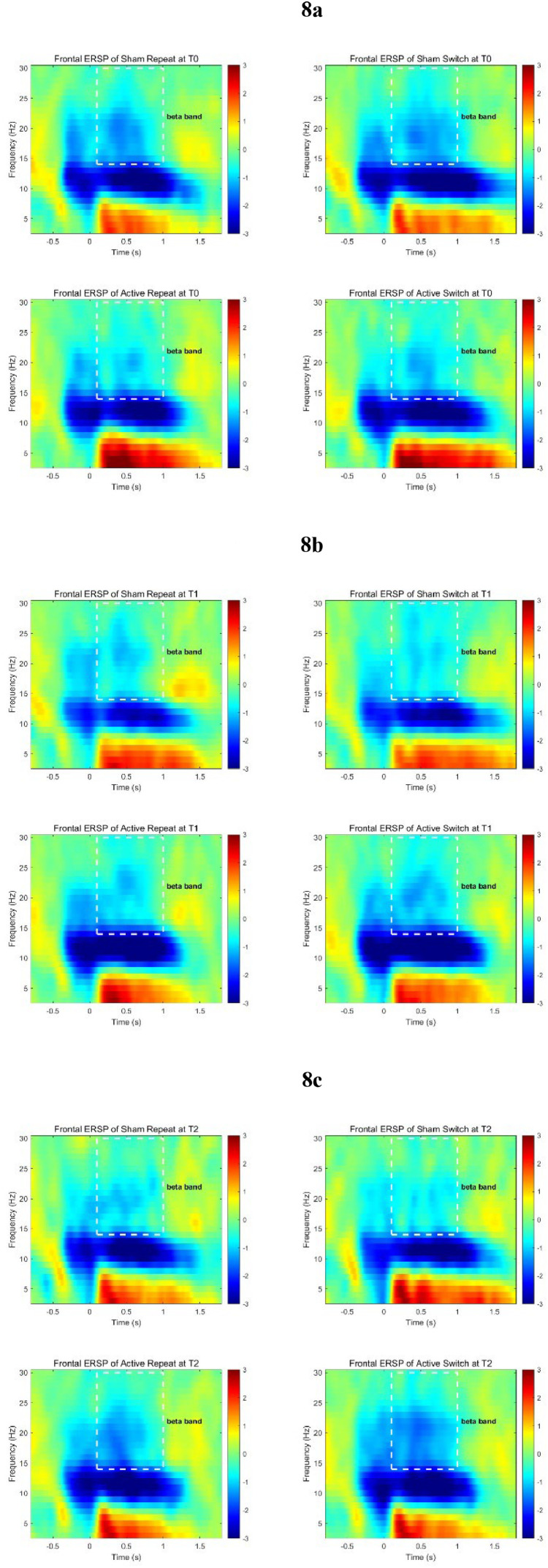
ERSP in the frontal region (F3, F4, Fz) a: T0; b: T1; c: T2.

**Fig. 9:**
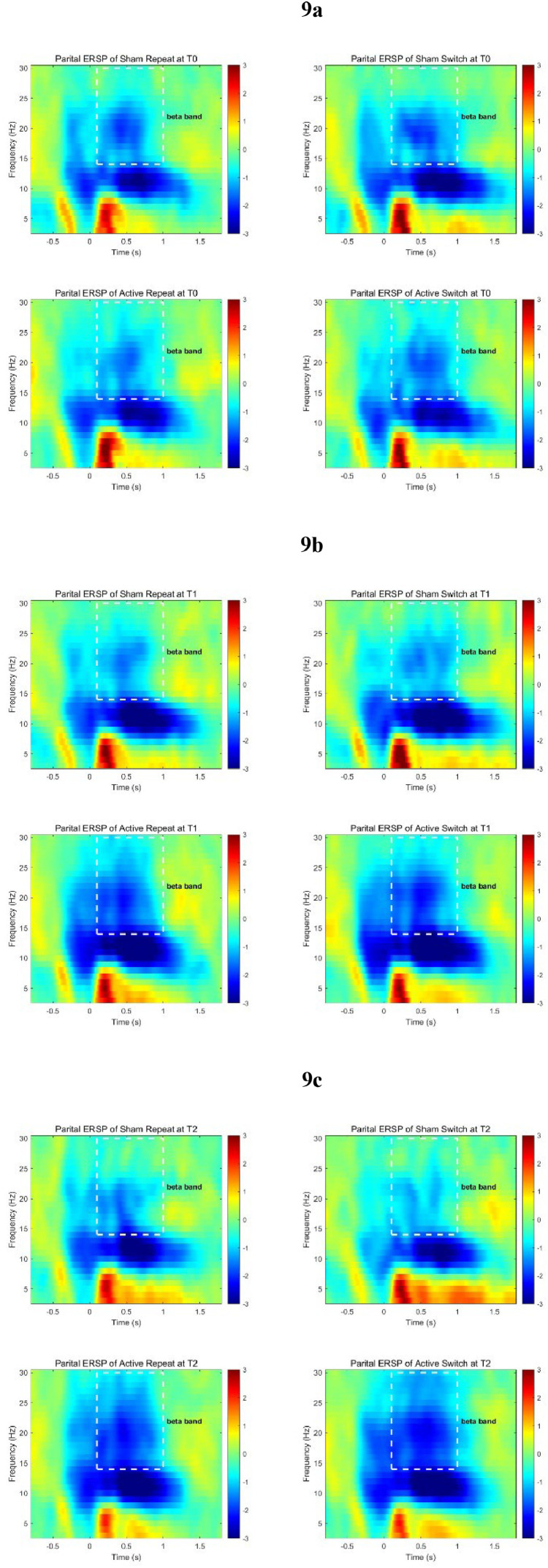
ERSP in the parietal region (P3, P4, Pz) a: T0; b: T1; c: T2.

ERSP graphs are shown in Figs. 8–9. The average power of all frequency bands and the comparison results are presented in Supplementary Material Tables 5–7. The mixed-design ANOVA revealed a significant interaction effect in the beta-band event-related desynchronization (ERD) at the parietal region under switch conditions (*P* < 0.05), and a marginally significant interaction effect in the beta-band at the frontal region (*P* = 0.06). The simple effects analysis indicated that at T2, under switch conditions, the Active group had a significantly lower ERD power in the beta band at the parietal region compared to the Sham group (*P* < 0.05). Under repeat conditions, the Active group had a lower ERD value in the beta band at the parietal region than the sham group, but the difference was only marginally significant (*P* = 0.07). Under switch conditions, the Active group had a larger ERD power in the beta band at the frontal region at T0 than at T1 and T2, with the differences being marginally significant (*P* < 0.08). At the parietal region, the beta band ERD power of Active group at T0 was significantly greater than at T2 (*P* < 0.05). Under repeat conditions, the beta band ERD power of Active group at the parietal region at T0 was significantly greater than at T1 and T2 (*P* < 0.05).

### Adverse events

1 participant (in the Active group) reported mild dizziness after the first session of rTMS and reported that it had resolved by the following morning when she got up; 2 participants (both in the Active group) reported tolerable mild headaches during the intervention, which disappeared immediately after the stimulation ended and did not reappear. No participant dropped out of the study due to adverse events.

### Additional intervention

After T2, 9 participants (Active group 7; Sham group 2) expressed their willingness to continue receiving the intervention. They all received an additional 10 sessions of rTMS intervention and did not report any adverse events.

## Discussions

In this study, we demonstrated that compared to sham stimulation, rTMS not only improved the sleep quality among undergraduates with insomnia symptoms but also aids in enhancing their cognitive flexibility. The PSQI and ISI scores in the Active group significantly decreased post-intervention, and the CFI score significantly increased; their performance in the N-L task was also superior to those of the Sham group post-intervention, indicating an enhanced ability to switch set to adapt to new rules.

The primary outcomes of this study indicated that rTMS could enhance the sleep quality of the participants, which aligned with our experimental expectations. However, despite the theoretical expectation that sham stimulation should have no effect, we observed a small but significant decrease in the ISI and PSQI scores of the Sham group following the intervention. Similar findings were also observed in the study by Wang et al. (2022). This “placebo effect” was also observed in the studies by B. Jiang et al. (2019) and Lin et al. (2023), that is, although the Sham group did not receive effective intervention, two weeks of sham stimulation also improved their self-assessment of sleep quality. However, placebo effect could not compare to the intervention effect of active stimulation, so at T1 and T2, the ISI and PSQI scores of the sham stimulation group were still significantly higher than those of the Active group.

Concerning the outcomes of the N-L task, we observed that, compared to the Sham group, at T2, the Active group exhibited a smaller decrease in RT, and its switch cost was even slightly higher than at T1. This observation may be attributed to the “ceiling effect”(Rock & Price, 2019) and the “practice effect”(Skaugset et al., 2016). Although rTMS intervention significantly enhanced the response speed of the Active group at T1, at T2, with the emergence of the ceiling effect and the waning of the rTMS intervention effect, the RT of the Active group tended to stabilize; however, under the impact of the practice effect, the response speed of the Sham group increased at both T1 and T2. Therefore, although at T1, the RT under switch conditions and switch cost of RT in the Active group were significantly lower than those of the Sham group; at T2, there were no significant differences in any RT indices between the two groups.

Nevertheless, the analysis of Acc might effectively supplement the RT results influenced by ceiling effect and practice effect: the Acc of the Active group exhibited an upward trend under the influence of rTMS intervention and practice effect, and the difference in Acc under different conditions (i.e., switch cost of Acc) also gradually diminished; whereas, due to being influenced solely by the practice effect, the Acc of the Sham group significantly increased at T1 and then slightly decreased at T2, thus the switch cost of Acc also showed no significant change. This result not only further verified the impact of rTMS on cognitive flexibility but also suggested a possibility that, despite the decrease in RT of the Sham group at T2, this might merely be due to their familiarity with the experimental task; this practice effect could not further increase their likelihood of making correct judgments when faced with rule changes.

The ERP results of this study support the above speculation: compared to T0, although the amplitudes of P2, N2, and P3 in both groups of participants decreased at T2, the P2 amplitudes in the frontal and parietal regions of the Active group were greater than those of the Sham group. The P2 component is primarily associated with the processing of perceptual information following stimulus presentation and early attention during task switching, and changes in its amplitude reflect the selective allocation of attentional resources by individuals(Du et al., 2022; Frober et al., 2021). Our results indicated that, although both groups of participants showed a decrease in P2 amplitude at T2 due to familiarity with the task; compared to the Sham group, the Active group could still allocate more attentional resources after stimulus presentation. Furthermore, no significant differences between groups were observed in N2 and P3 components, our results provided some clues to the impact of rTMS on neurophysiological activity as well. In the task switching paradigm, N2 primarily reflects an individual's monitoring of conflicting rules and their ability to inhibit control of previous task(J. Chen et al., 2024); P3 involves an individual's working memory updating and target detection, and its amplitude is considered to be related to the integration of task-related information and stimulus evaluation(Berchio et al., 2023; Feng & Feng, 2022). We observed that the N2 amplitude in the frontal region of the Sham group showed a decreasing trend at different time points, which might reflect that the practice effect had enhanced their ability to inhibit previous conflicting rules, thus manifesting as a decrease in RT. Moreover, Gajewski et al. (2018) and J. Chen et al. (2022) both pointed out that in the task switching paradigm, as the complexity of the task increased, the amplitude of frontal P3 would decrease. In this study, at both T1 and T2, the amplitudes of various components of P3 in the frontal region of the Active group were greater than those of the Sham group (though the differences were not statistically significant), which might indicate that following active rTMS intervention, participants perceived the task complexity to be lower than those who received sham stimulation, thereby resulting in a decrease in RT and an increase in Acc.

The analysis of ERSP power in this study suggested changes in the frontal and parietal region after rTMS intervention deeply. The beta band ERD reflects the level of cortical excitability, where a decrease in ERD power corresponds to an increase in cortical excitability levels(Olyaei et al., 2022). And the beta band ERD in the parietal region is also related to the integration of cognitive and motor activities(Ozga et al., 2019). The findings of this study revealed that after the rTMS intervention, the Active group exhibited a decreasing trend in ERD in both the frontal and parietal regions, with the parietal region showing a more significant change. This could indicate that the rTMS intervention enhanced the neural network connectivity between the frontal and parietal regions of the participants, leading to more efficient information processing in the brain. In addition, this phenomenon also helps to explain the results of the behavioral task at T2: Although the difference in RT between the two groups was not significant due to the ceiling effect, the Active group demonstrated heightened cortical excitability and superior capacity to merge cognitive processing with motor execution. This enhancement likely contributed to their increased ability to make accurate choices (resulting in higher Acc).

To our knowledge, it was the first study to explore the impact of rTMS on cognitive flexibility among individuals with insomnia symptoms. Although researchers have already recognized the value of rTMS in neuroenhancement(Antal et al., 2022), most existing studies using rTMS to enhance cognitive abilities of participants tend to opt for HF-rTMS rather than LF-rTMS (Ameis et al., 2020; Chu et al., 2021; Y. Jiang et al., 2019). This may be due to the general belief that LF-rTMS often produces long-term depression (LTD) effects, while HF-rTMS mainly produces long-term potentiation (LTP) effects(Suppa et al., 2022). However, according to the hyperarousal model of insomnia(Dressle & Riemann, 2023), due to the hyperactivation of the hypothalamic-pituitary-adrenal axis (HPA axis) and the hyperarousal of the PFC, the daytime functioning of individuals with insomnia symptoms would be impaired. Therefore, employing HF-rTMS on individuals with insomnia symptoms could potentially worsen their sleep quality and was not beneficial for enhancing cognitive flexibility. On the contrary, LF-rTMS may alleviate the functional abnormalities of the PFC and even the FPN in individuals with insomnia symptoms to some extent by inhibiting the hyperarousal of the cortex(Sutcubasi et al., 2024), and/or by affecting the levels of glutamate/GABA within these structures(Godfrey et al., 2021). On the other hand, due to its frequency being close to the natural slow wave frequency band (0.5–4 Hz), LF-rTMS has a positive effect on promoting slow wave sleep (DiNuzzo et al., 2022). And according to the synaptic homeostasis hypothesis (Tononi & Cirelli, 2006), sufficient slow wave sleep could enhance functional connectivity between the PFC and subcortical structures like the thalamus and striatum (Lanfranco et al., 2024), and protects the synaptic plasticity of PFC by reducing the levels of pro-inflammatory factors and oxidative stress markers (Embang et al., 2025), thereby enhancing cognitive flexibility. In addition, excessive norepinephrine (NE) levels would impair the ability to inhibit irrelevant stimuli (Yu et al., 2024), while good sleep quality helps stabilize NE levels (Van Someren, 2021). Therefore, by a combination of these possible mechanisms, LF-rTMS could promote cognitive flexibility on the basis of improving their sleep quality.

This study differed from previous researches in several aspects: we used self-rated questionnaires and behavioral tasks in combination to comprehensively assess the improvement of cognitive flexibility in participants; we also applied ERP to preliminarily explore the potential mechanisms of rTMS affecting cognitive flexibility. In addition, we set an 8-weeks follow-up test after the intervention to observe the effect of rTMS intervention. Additionally, 27.3 % (9/33) of the participants, especially 41.2 % (7/17) of the intervention group, were willing to continue receiving intervention after the trial ended, which reflected the high acceptance and compliance of rTMS among individuals with insomnia, suggesting not only the safety but also the feasibility of exploring rTMS to improve individuals’ cognitive flexibility with insomnia in a larger population.

At the same time, we should also acknowledge some limitations in this study. Firstly, this study was a single-center study, and all participants were from the same university, which might affect the generalizability of the conclusions. Secondly, although the sample size met the requirements of a prospective study, the relatively small sample size still affected our interpretation of the results. Thirdly, while this study included neuropsychological assessments, behavioral data, ERPs, and neural oscillations, it would benefit from the addition of other neuroimaging techniques like functional near-infrared spectroscopy or multi-voxel pattern analysis. In addition, owing to the management of the university, we were unable to use polysomnography to record the physiological changes of the participants during sleep, which could further reveal how insomnia affects cognitive flexibility and how rTMS intervention influences this relationship.

## Conclusions

This study indicates the feasibility of using rTMS to enhance cognitive flexibility among undergraduates with insomnia symptoms. Future researchers should adopt more comprehensive approaches (e.g., combining other neuroimaging techniques like fMRI or measuring the levels of neurotransmitters and hormones) to explore the application prospects of this technology in different populations and the underlying neurobiological mechanisms.

## CRediT authorship contribution statement

**Muyu Chen:** Conceptualization, Formal analysis, Investigation, Methodology, Software, Visualization, Writing – original draft, Writing – review & editing; **Jun Jiang**: Investigation, Software, Supervision; **Han Chen**: Investigation, Methodology, Writing - Review & Editing; **Xinyu Liu:** Formal analysis, Investigation; **Xinpeng Zhang**: Formal analysis, Investigation; **Li Peng:** Conceptualization, Funding acquisition, Methodology, Project administration, Resources, Supervision, Writing – review & editing.

## Declaration of generative AI and AI-assisted technologies in the writing process

The authors did not use any generative AI or AI-assisted technologies in the writing process.

## Funding sources

This study was funded by Chongqing Municipal Education Commission Science and Technology Research Project (KJQN202312806) and National College Students Innovation and Entrepreneurship Training Program (202490031029). The funding bodies had no role in study design, data collection and analysis, interpretation of data, or in writing the manuscript.

## Declaration of competing interest

The authors have no conflicts of interest to declare.

## Appendix Supplementary materials

Image, application 1

## References

[bib0001] Ameis S.H., Blumberger D.M., Croarkin P.E., Mabbott D.J., Lai M.C., Desarkar P., Daskalakis Z.J. (2020). Treatment of Executive Function deficits in autism spectrum disorder with repetitive transcranial magnetic stimulation: A double-blind, sham-controlled, pilot trial. Brain Stimulation.

[bib0002] American Academy of Sleep Medicine (2014).

[bib0003] Anastasiades P.G., de Vivo L., Bellesi M., Jones M.W. (2022). Adolescent sleep and the foundations of prefrontal cortical development and dysfunction. Progress in Neurobiology.

[bib0004] Antal A., Luber B., Brem A.K., Bikson M., Brunoni A.R., Cohen Kadosh R., Paulus W. (2022). Non-invasive brain stimulation and neuroenhancement. Clinical Neurophysiology Practice.

[bib0005] Bai Y., Xuan J., Jia S., Ziemann U. (2023). TMS of parietal and occipital cortex locked to spontaneous transient large-scale brain states enhances natural oscillations in EEG. Brain Stimulation.

[bib0006] Ballesio A., Cerolini S., Vacca M., Lucidi F., Lombardo C. (2020). Insomnia symptoms moderate the relationship between perseverative cognition and backward inhibition in the task-switching paradigm. Frontiers in Psychology.

[bib0007] Bastien C.H., Vallieres A., Morin C.M. (2001). Validation of the Insomnia Severity Index as an outcome measure for insomnia research. Sleep Medicine.

[bib0008] Batzikosta A., Moraitou D., Steiropoulos P., Papantoniou G., Kougioumtzis G.A., Katsouri I.G., Tsolaki M. (2024). The relationships of specific cognitive control abilities with objective and subjective sleep parameters in mild cognitive impairment: Revealing the association between cognitive planning and sleep duration. Brain Sciences.

[bib0009] Berchio C., Annen L.C., Bouamoud Y., Micali N. (2023). Temporal dynamics of cognitive flexibility in adolescents with anorexia nervosa: A high-density EEG study. European Journal of Neuroscience.

[bib0010] Brooks S.J., Katz E.S., Stamoulis C. (2022). Shorter duration and lower quality sleep have widespread detrimental effects on developing functional brain networks in early adolescence. Cerebral Cortex Communications.

[bib0011] Buysse D.J., Reynolds C.F., Monk T.H., Berman S.R., Kupfer D.J. (1989). The pittsburgh sleep quality index: A new instrument for psychiatric practice and research. Psychiatry Research.

[bib0012] Chen J., Kwok A.P.K., Li Y. (2024). Postural control and cognitive flexibility in skilled athletes: Insights from dual-task performance and event-related potentials. Brain research Bulletin.

[bib0013] Chen J., Wu S., Li F. (2022). Cognitive neural mechanism of backward inhibition and deinhibition: A review. Frontiers in Behavioral Neuroscience.

[bib0014] Chen M., Zhang X., Liu X., Chen Y., Liu R., Peng L., Li M. (2024). The association between insomnia symptoms and cognitive flexibility among undergraduates: An event-related potential study. Sleep Medicine.

[bib0015] Chen Q., Zhao J., Gu H., Li X. (2022). Inhibitory control of emotional interference in deaf children: Evidence from event-related potentials and event-related spectral perturbation analysis. Frontiers in Psychiatry.

[bib0016] Chen Y., Wang Y., Wang S., Zhang M., Wu N. (2021). Self-reported sleep and executive function in early primary school children. Frontiers in Psychology.

[bib0017] Chen Y., Zhang Y., Yu G. (2022). Prevalence of mental health problems among college students in mainland China from 2010 to 2020: A meta-analysis. Advances in Psychological Science.

[bib0018] Cheng W., Rolls E.T., Ruan H., Feng J. (2018). Functional connectivities in the brain that mediate the association between depressive problems and sleep quality. JAMA Psychiatry.

[bib0019] Chu C.S., Li C.T., Brunoni A.R., Yang F.C., Tseng P.T., Tu Y.K., Liang C.S. (2021). Cognitive effects and acceptability of non-invasive brain stimulation on Alzheimer's disease and mild cognitive impairment: A component network meta-analysis. Journal of Neurology, Neurosurgery and Psychiatry.

[bib0020] Dajani D.R., Uddin L.Q. (2015). Demystifying cognitive flexibility: Implications for clinical and developmental neuroscience. Trends in Neurosciences.

[bib0021] Dennis J.P., Wal J.S.V. (2010). The cognitive flexibility inventory: Instrument development and estimates of reliability and validity. Cognitive Therapy and Research.

[bib0022] Diamond A. (2013). Executive functions. Annual Review of Psychology.

[bib0023] DiNuzzo M., Mangia S., Giove F. (2022). Manipulations of sleep-like slow-wave activity by noninvasive brain stimulation. Journal of Neuroscience Research.

[bib0024] Dressle R.J., Riemann D. (2023). Hyperarousal in insomnia disorder: Current evidence and potential mechanisms. Journal of Sleep Research.

[bib0025] Du M., Peng Y., Li Y., Zhu Y., Yang S., Li J., Zhang M. (2022). Effect of trait anxiety on cognitive flexibility: Evidence from event-related potentials and resting-state EEG. Biological Psychology.

[bib0026] Embang J.E.G., Tan Y.H.V., Ng Y.X., Loyola G.J.P., Wong L.W., Guo Y., Dong Y. (2025). Role of sleep and neurochemical biomarkers in synaptic plasticity related to neurological and psychiatric disorders: A scoping review. Journal of Neurochemistry.

[bib0027] Feng X., Feng C.Z. (2022). The effect of cognitive flexibility on probabilistic category learning. Acta Psychologica Sinica.

[bib0028] Friedman N.P., Robbins T.W. (2022). The role of prefrontal cortex in cognitive control and executive function. Neuropsychopharmacology : Official publication of the American College of Neuropsychopharmacology.

[bib0029] Frober K., Jurczyk V., Mendl J., Dreisbach G. (2021). Investigating anticipatory processes during sequentially changing reward prospect: An ERP study. Brain and Cognition.

[bib0030] Gajewski P.D., Ferdinand N.K., Kray J., Falkenstein M. (2018). Understanding sources of adult age differences in task switching: Evidence from behavioral and ERP studies. Neuroscience and Biobehavioral Reviews.

[bib0031] Ganesan K., Steinbeis N. (2022). Development and plasticity of executive functions: A value-based account. Current Opinion in Psychology.

[bib0032] Godfrey K.E.M., Muthukumaraswamy S.D., Stinear C.M., Hoeh N. (2021). Effect of rTMS on GABA and glutamate levels in treatment-resistant depression: An MR spectroscopy study. Psychiatry research. Neuroimaging.

[bib0033] Guarana C.L., Ryu J.W., O'Boyle E.H., Lee J., Barnes C.M (2021). Sleep and self-control: A systematic review and meta-analysis. Sleep Medicine Reviews.

[bib0034] Hernandez-Sauret A., Martin de la Torre O., Redolar-Ripoll D. (2024). Use of transcranial magnetic stimulation (TMS) for studying cognitive control in depressed patients: A systematic review. Cognitive, Affective & Behavioral Neuroscience.

[bib0035] Jiang B., He D., Guo Z., Mu Q., Zhang L. (2019). Efficacy and placebo response of repetitive transcranial magnetic stimulation for primary insomnia. Sleep Medicine.

[bib0036] Jiang Y., Guo Z., Xing G., He L., Peng H., Du F., Mu Q. (2019). Effects of high-frequency transcranial magnetic stimulation for cognitive deficit in schizophrenia: A meta-analysis. Frontiers in Psychiatry.

[bib0037] Kim S.Y., Lee K.H., Lee H., Jeon J.E., Kim S., Lee M.H., Lee Y.J. (2022). Neural activation underlying emotional interference of cognitive control in rotating shift workers: Moderating effects of the prefrontal cortex response on the association between sleep disturbance and depressive symptoms. Sleep.

[bib0038] King N., Pickett W., Keown-Stoneman C.D.G., Miller C.B., Li M., Duffy A. (2023). Changes in sleep and the prevalence of probable insomnia in undergraduate university students over the course of the COVID-19 pandemic: Findings from the U-Flourish cohort study. BJPsych Open.

[bib0039] Kong J., Zhou L., Li X., Ren Q. (2023). Sleep disorders affect cognitive function in adults: An overview of systematic reviews and meta-analyses. Sleep and Biological Rhythms.

[bib0040] Krone L.B., Feher K.D., Rivero T., Omlin X. (2023). Brain stimulation techniques as novel treatment options for insomnia: A systematic review. Journal of Sleep Research.

[bib0041] Lanfranco R.C., Dos Santos Sousa F., Wessel P.M., Rivera-Rei A., Bekinschtein T.A., Lucero B., Huepe D. (2024). Slow-wave brain connectivity predicts executive functioning and group belonging in socially vulnerable individuals. Cortex; A Journal Devoted to the Study of the Nervous System and Behavior.

[bib0042] Lanza G., Cantone M., Arico D., Lanuzza B., Cosentino F.I.I., Paci D., Ferri R. (2018). Clinical and electrophysiological impact of repetitive low-frequency transcranial magnetic stimulation on the sensory-motor network in patients with restless legs syndrome. Therapeutic Advances in Neurological Disorders.

[bib0043] Lanza G., Fisicaro F., Cantone M., Pennisi M., Cosentino F.I.I., Lanuzza B., Ferri R. (2023). Repetitive transcranial magnetic stimulation in primary sleep disorders. Sleep Medicine Reviews.

[bib0044] Lefaucheur J.P. (2019). Transcranial magnetic stimulation. Handbook of Clinical Neurology.

[bib0045] Li S., Hu S., Zhou H., Chen J., Dong W., Wang H., Huang M. (2024). Interpretation of association standard of Operating specifications for repetitive transcranial Magnetic stimulation in clinical applications on psychiatric disorders [Article]. Chinese Journal of Psychiatry.

[bib0046] Lin W.C., Chen M.H., Liou Y.J., Tu P.C., Chang W.H., Bai Y.M., Su T.P. (2023). Effect of low-frequency repetitive transcranial magnetic stimulation as adjunctive treatment for insomnia patients under hypnotics: A randomized, double-blind, sham-controlled study. Journal of the Chinese Medical Association: JCMA.

[bib0047] Liu X., Tang M., Hu L., Wang A. (1996). Reliability and validity of the Pittsburgh Sleep Quality Index. Chinese Journal of Psychiatry.

[bib0048] Lu T., Li Y., Xia P., Zhang G., Wu D. (2014). Analysis on reliability and validity of the Pittsburgh sleep quality index. Chongqing Medical Journal.

[bib0049] Morin C.M., Belleville G., Belanger L., Ivers H. (2011). The Insomnia Severity Index: Psychometric indicators to detect insomnia cases and evaluate treatment response. Sleep.

[bib0050] Olyaei G., Khanmohammadi R., Talebian S., Hadian M.R., Bagheri H., Najafi M. (2022). The effect of exergaming on cognition and brain activity in older adults: A motor- related cortical potential study. Physiology & Behavior.

[bib0051] Ozga W.K., Zapala D., Wierzgala P., Augustynowicz P., Porzak R., Wojcik G.M. (2019). Acoustic neurofeedback increases beta ERD during mental rotation task. Applied Psychophysiology and Biofeedback.

[bib0052] Perlis M.L., Posner D., Riemann D., Bastien C.H., Teel J., Thase M. (2022). Insomnia. Lancet (London, England).

[bib0053] Pitcher D., Parkin B., Walsh V. (2021). Transcranial magnetic stimulation and the understanding of behavior. Annual Review of Psychology.

[bib0054] Riemann D., Espie C.A., Altena E., Arnardottir E.S., Baglioni C., Bassetti C.L.A., Spiegelhalder K. (2023). The European Insomnia Guideline: An update on the diagnosis and treatment of insomnia 2023. Journal of Sleep Research.

[bib0055] Rock D., Price I.R. (2019). Identifying culturally acceptable cognitive tests for use in remote northern Australia. BMC Psychology.

[bib0056] Romero-Ferreiro V., Rodriguez-Gomez P., Pozo M.A., Moreno E.M. (2022). Can you change your mind? An ERP study of cognitive flexibility and new evidence integration. Biological Psychology.

[bib0057] Schmitter-Edgecombe M., Langill M. (2006). Costs of a predictable switch between simple cognitive tasks following severe closed-head injury. Neuropsychology.

[bib0058] Siqi-Liu A., Egner T., Woldorff M.G. (2022). Neural dynamics of context-sensitive adjustments in cognitive flexibility. Journal of Cognitive Neuroscience.

[bib0059] Skaugset L.M., Farrell S., Carney M., Wolff M., Santen S.A., Perry M., Cico S.J. (2016). Can you multitask? Evidence and limitations of task switching and multitasking in emergency medicine. Annals of Emergency Medicine.

[bib0060] Sun N., He Y., Wang Z., Zou W., Liu X. (2021). The effect of repetitive transcranial magnetic stimulation for insomnia: A systematic review and meta-analysis. Sleep Medicine.

[bib0061] Suppa A., Asci F., Guerra A. (2022). Transcranial magnetic stimulation as a tool to induce and explore plasticity in humans. Handbook of Clinical Neurology.

[bib0062] Sutcubasi B., Bayram A., Metin B., Demiralp T. (2024). Neural correlates of approach-avoidance behavior in healthy subjects: Effects of low-frequency repetitive transcranial magnetic stimulation (rTMS) over the right dorsolateral prefrontal cortex. International Journal of Psychophysiology: Official Journal of the In Ternational Organization of Psychophysiology.

[bib0063] Sutton E.L. (2021). Insomnia. Annals of Internal Medicine.

[bib0064] Tononi G., Cirelli C. (2006). Sleep function and synaptic homeostasis. Sleep Medicine Reviews.

[bib0065] Van Someren E.J.W. (2021). Brain mechanisms of insomnia: New perspectives on causes and consequences. Physiological Reviews.

[bib0066] Wang L., Zhang J., Guo C., He J., Zhang S., Wang Y., Rong P. (2022). The efficacy and safety of transcutaneous auricular vagus nerve stimulation in patients with mild cognitive impairment: A double blinded randomized clinical trial. Brain Stimulation.

[bib0067] Wang Y. (2024). Chinese guideline for diagnosis and treatment of insomnia (2023) [Article]. Chinese Journal of Neurology.

[bib0068] World Health Organization (2021). https://icd.who.int/.

[bib0069] Xu M., Nikolin S., Samaratunga N., Chow E.J.H., Loo C.K., Martin D.M. (2023). Cognitive effects following offline high-frequency repetitive transcranial magnetic stimulation (HF-rTMS) in healthy populations: A systematic review and meta-analysis [Review; Early Access]. Neuropsychology Review.

[bib0070] Yao Z.F., Yang M.H., Hwang K., Hsieh S. (2020). Frontoparietal structural properties mediate adult life span differences in executive function. Scientific Reports.

[bib0071] Yu S., Konjusha A., Ziemssen T., Beste C. (2024). Inhibitory control in WM gate-opening: Insights from alpha desynchronization and norepinephrine activity under atDCS stimulation. Neuroimage.

[bib0072] Yuan P., Raz N. (2014). Prefrontal cortex and executive functions in healthy adults: A meta-analysis of structural neuroimaging studies. Neuroscience and Biobehavioral Reviews.

[bib0073] Zhang X., Feng S., Yang X., Peng Y., Du M., Zhang R., Zhang M. (2024). Neuroelectrophysiological alteration associated with cognitive flexibility after 24 h sleep deprivation in adolescents. Consciousness and Cognition.

[bib0074] Zhuo B., Zhu M., Cao B., Li F. (2021). More change in task repetition, less cost in task switching: Behavioral and event-related potential evidence. European Journal of Neuroscience.

