# Supplementary material for: The effects of transcranial magnetic stimulation on cognitive flexibility among undergraduates with insomnia symptoms: A prospective, single-blind, randomized control trial
Source: Int J Clin Health Psychol. 2025 Apr 14;25(2):100567. doi: 10.1016/j.ijchp.2025.100567 (PMC12019015; doi:10.1016/j.ijchp.2025.100567)
Supplement: Supplementary file 1 [file mmc1.docx]

**Table1 Correlations between changes in insomnia and cognitive flexibility**

|  | M±SD | δ1_PSQI | δ1_ISI | δ1_CFI |
| --- | --- | --- | --- | --- |
| δ1_PSQI | -3.34±2.87 | - | - | - |
| δ1_ISI | -6.86±6.07 | 0.62^**^ | - | - |
| δ1_CFI | 5.00±16.77 | -0.54^**^ | -0.61^**^ | - |
|  | M±SD | δ2_PSQI | δ2_ISI | δ2_CFI |
| δ2_PSQI | -2.86±3.84 | - | - | - |
| δ2_ISI | -6.21±6.01 | 0.68^***^ | - | - |
| δ2_CFI | 1.52±17.32 | -0.45^*^ | -0.68^***^ | - |
|  | M±SD | δ3_PSQI | δ3_ISI | δ3_CFI |
| δ3_PSQI | 0.48±2.91 | - | - | - |
| δ3_ISI | 0.66±4.50 | 0.66^***^ | - | - |
| δ3_CFI | -3.48±8.69 | -0.01 | 0.01 | - |

Notes: ^*^: *P*<0.05; ^***^: *P*<0.001; δ1: T1-T0; δ2: T2-T0; δ3: T2-T1

**Table2 Comarisons of P2 components(μV)**

| Regions | Conditions | Time | Active group | Sham group | *t* |  | *F* |  |
| --- | --- | --- | --- | --- | --- | --- | --- | --- |
|  |  |  | (*n*=15) | (*n*=14) |  | Interaction | Time | Group |
| Frontal | Repeat | T0 | 5.27±2.49 | 5.61±4.07 | -0.27 | 1.90 | 19.09^***^ | 0.62 |
|  |  | T1 | 5.79±2.39 | 4.94±3.76 | 0.74 |  |  |  |
|  |  | T2 | 3.47±2.32 | 1.80±2.35 | 1.93 |  |  |  |
|  |  | Multiple comparison | T0>T2^#^, T1>T2 | T0>T2, T1>T2 | - |  |  |  |
|  | Switch | T0 | 5.28±1.87 | 6.04±3.38 | -0.76 | 4.31^*^ | 20.29^***^ | 1.06 |
|  |  | T1 | 5.71±2.09 | 4.74±3.56 | 0.89 |  |  |  |
|  |  | T2 | 3.78±2.28 | 1.61±1.90 | 2.77^*^ |  |  |  |
|  |  | Multiple comparison | T0>T2^#^, T1>T2 | T0>T2, T1>T2 | - |  |  |  |
| Parietal | Repeat | T0 | 8.16±4.49 | 7.07±3.59 | 0.72 | 0.18 | 10.23^***^ | 1.83 |
|  |  | T1 | 6.29±3.91 | 5.47±3.86 | 0.57 |  |  |  |
|  |  | T2 | 4.70±2.87 | 2.89±2.48 | 1.81 |  |  |  |
|  |  | Multiple comparison | T0>T2, T1>T2^#^ | T0>T2 | - |  |  |  |
|  | Switch | T0 | 7.71±4.36 | 7.74±3.69 | -0.17 | 0.57 | 11.39^***^ | 0.74 |
|  |  | T1 | 6.78±4.28 | 6.00±3.78 | 0.52 |  |  |  |
|  |  | T2 | 4.77±2.44 | 3.07±2.40 | 1.89 |  |  |  |
|  |  | Multiple comparison | T0>T2^#^ | T0>T1^#^, T1>T2^#^,  T0>T2 | - |  |  |  |

Notes: ^*^: *P*<0.05; ^***^: *P*<0.001; ^#^: *P*<0.05 and *P*_adj>0.05

**Table3 Comarisons of N2 components(μV)**

| Regions | Conditions | Time | Active group | Sham group | *t* |  | *F* |  |
| --- | --- | --- | --- | --- | --- | --- | --- | --- |
|  |  |  | (*n*=15) | (*n*=14) |  | Interaction | Time | Group |
| Frontal | Repeat | T0 | 3.15±4.03 | 5.11±4.38 | -1.26 | 1.81 | 6.82^**^ | 0.28 |
|  |  | T1 | 3.06±3.50 | 3.59±4.66 | -0.34 |  |  |  |
|  |  | T2 | 1.98±2.93 | 1.39±3.35 | 0.51 |  |  |  |
|  |  | Multiple comparison | - | T0>T2, T1>T2 | - |  |  |  |
|  | Switch | T0 | 2.45±3.99 | 4.79±4.25 | -1.53 | 4.39^*^ | 9.30^***^ | 0.23 |
|  |  | T1 | 2.51±3.37 | 2.93±3.58 | -0.33 |  |  |  |
|  |  | T2 | 1.65±2.57 | 0.41±2.48 | 1.32 |  |  |  |
|  |  | Multiple comparison | - | T0>T1^#^, T1>T2,  T0>T2 | - |  |  |  |
| Parietal | Repeat | T0 | 6.08±2.60 | 6.63±2.85 | -0.54 | 0.18 | 19.73^***^ | 0.01 |
|  |  | T1 | 4.93±3.06 | 4.58±2.97 | 0.31 |  |  |  |
|  |  | T2 | 1.73±3.90 | 1.73±3.73 | 0.00 |  |  |  |
|  |  | Multiple comparison | T0>T2, T1>T2 | T0>T1^#^, T1>T2^#^,  T0>T2 | - |  |  |  |
|  | Switch | T0 | 5.96±2.85 | 7.97±3.03 | -1.84 | 1.10 | 34.23^***^ | 0.87 |
|  |  | T1 | 5.26±3.31 | 5.79±2.98 | -0.45 |  |  |  |
|  |  | T2 | 1.18±3.66 | 1.05±3.62 | 0.10 |  |  |  |
|  |  | Multiple comparison | T0>T2, T1>T2 | T0>T1^#^, T1>T2,  T0>T2 | - |  |  |  |

Notes: ^*^: *P*<0.05; ^**^: *P*<0.01; ^***^: *P*<0.001; ^#^: *P*<0.05 and *P*_adj>0.05

**Table4 Comarisons of P3 components(μV)**

| Regions | Conditions | Time | Active group | Sham group | *t* |  | *F* |  |
| --- | --- | --- | --- | --- | --- | --- | --- | --- |
|  |  |  | (*n*=15) | (*n*=14) |  | Interaction | Time | Group |
| Frontal | Repeat | T0 | 3.63±3.82 | 3.80±3.83 | -0.12 | 0.51 | 6.45^**^ | 0.27 |
|  |  | T1 | 3.53±3.38 | 2.63±3.66 | 0.68 |  |  |  |
|  |  | T2 | 2.03±3.00 | 1.10±2.89 | 0.85 |  |  |  |
|  |  | Multiple comparison | T0>T2^#^ | T0>T2, T1>T2^#^ | - |  |  |  |
|  | Switch | T0 | 2.29±3.49 | 3.09±3.37 | -0.63 | 1.23 | 5.38^**^ | 0.10 |
|  |  | T1 | 2.06±3.66 | 1.99±3.13 | 0.06 |  |  |  |
|  |  | T2 | 1.32±2.73 | 0.32±2.56 | 1.02 |  |  |  |
|  |  | Multiple comparison | - | T0>T2, T1>T2^#^ | - |  |  |  |
| Parietal | Repeat | T0 | 7.11±3.00 | 6.74±3.06 | 0.33 | 0.05 | 26.87^***^ | 0.46 |
|  |  | T1 | 5.17±3.47 | 4.37±2.16 | 0.73 |  |  |  |
|  |  | T2 | 2.44±3.89 | 1.84±2.56 | 0.49 |  |  |  |
|  |  | Multiple comparison | T0>T1^#^, T1>T2,  T0>T2 | T0>T1>T2 | - |  |  |  |
|  | Switch | T0 | 6.24±3.24 | 7.41±2.99 | -1.01 | 0.65 | 45.08^***^ | 0.29 |
|  |  | T1 | 5.06±3.63 | 5.52±2.15 | -0.41 |  |  |  |
|  |  | T2 | 1.31±3.15 | 1.07±2.51 | 0.22 |  |  |  |
|  |  | Multiple comparison | T0>T2, T1>T2 | T0>T1^#^, T1>T2,  T0>T2 | - |  |  |  |

Notes: ^**^: *P*<0.01; ^***^: *P*<0.001; ^#^: *P*<0.05 and *P*_adj>0.05

**Table5 Comparisons of θ band (100-400 ms) ERSP (dB)**

| Regions | Conditions | Time | Active group | Sham group | *t* |  | *F* |  |
| --- | --- | --- | --- | --- | --- | --- | --- | --- |
|  |  |  | (*n*=15) | (*n*=14) |  | Interaction | Time | Group |
| Frontal | Repeat | T0 | 1.55±0.95 | 1.14±0.82 | 1.22 | 1.00 | 0.43 | 0.22 |
|  |  | T1 | 1.34±0.88 | 1.26±0.72 | 1.27 |  |  |  |
|  |  | T2 | 1.12±1.09 | 1.24±0.90 | -0.33 |  |  |  |
|  |  | Multiple comparison | - | - |  |  |  |  |
|  | Switch | T0 | 1.19±0.90 | 1.04±0.66 | 0.53 | 1.01 | 0.48 | 0.00 |
|  |  | T1 | 1.12±0.89 | 0.96±0.50 | 0.58 |  |  |  |
|  |  | T2 | 1.07±1.00 | 1.37±1.03 | -0.79 |  |  |  |
|  |  | Multiple comparison | - | - |  |  |  |  |
| Parietal | Repeat | T0 | 2.16±1.39 | 2.06±1.38 | 0.19 | 0.70 | 2.56 | 0.26 |
|  |  | T1 | 1.86±1.23 | 2.12±0.73 | -0.68 |  |  |  |
|  |  | T2 | 1.39±1.63 | 1.83±1.07 | -0.86 |  |  |  |
|  |  | Multiple comparison | T0>T2 | - |  |  |  |  |
|  | Switch | T0 | 1.96±1.14 | 2.33±1.31 | -0.81 | 0.10 | 2.36 | 1.51 |
|  |  | T1 | 1.77±1.45 | 2.29±1.11 | -1.07 |  |  |  |
|  |  | T2 | 1.43±1.25 | 1.98±1.08 | -1.26 |  |  |  |
|  |  | Multiple comparison | - | - |  |  |  |  |

**Table6 Comparisons of α band (400-1000 ms) ERSP (dB)**

| Regions | Conditions | Time | Active group | Sham group | *t* |  | *F* |  |
| --- | --- | --- | --- | --- | --- | --- | --- | --- |
|  |  |  | (*n*=15) | (*n*=14) |  | Interaction | Time | Group |
| Frontal | Repeat | T0 | -2.42±2.10 | -2.69±1.90 | 0.36 | 0.90 | 0.23 | 0.13 |
|  |  | T1 | -2.65±1.98 | -1.99±1.49 | -1.00 |  |  |  |
|  |  | T2 | -2.61±2.42 | -2.32±2.00 | -0.35 |  |  |  |
|  |  | Multiple comparison | - | - |  |  |  |  |
|  | Switch | T0 | -2.56±2.43 | -2.70±1.91 | 0.17 | 0.56 | 0.13 | 0.17 |
|  |  | T1 | -2.76±2.16 | -2.12±1.51 | -0.91 |  |  |  |
|  |  | T2 | -2.69±2.59 | -2.32±2.35 | -0.40 |  |  |  |
|  |  | Multiple comparison | - | - |  |  |  |  |
| Parietal | Repeat | T0 | -2.35±1.64 | -2.23±1.62 | -0.18 | 0.16 | 1.35 | 0.80 |
|  |  | T1 | -2.80±1.77 | -2.77±1.92 | -0.03 |  |  |  |
|  |  | T2 | -2.79±2.45 | -2.45±1.88 | -0.43 |  |  |  |
|  |  | Multiple comparison | - | - |  |  |  |  |
|  | Switch | T0 | -2.22±1.79 | -2.58±2.01 | 0.51 | 0.50 | 1.37 | 0.00 |
|  |  | T1 | -2.89±2.00 | -2.96±2.00 | 0.09 |  |  |  |
|  |  | T2 | -2.75±2.59 | -2.47±2.25 | -0.31 |  |  |  |
|  |  | Multiple comparison | - | - |  |  |  |  |

**Table7 Comparisons of β band (100-1000 ms) ERSP (dB)**

| Regions | Conditions | Time | Active group | Sham group | *t* |  | *F* |  |
| --- | --- | --- | --- | --- | --- | --- | --- | --- |
|  |  |  | (*n*=15) | (*n*=14) |  | Interaction | Time | Group |
| Frontal | Repeat | T0 | -0.92±0.77 | -1.01±0.64 | 0.35 | 2.03 | 0.26 | 0.74 |
|  |  | T1 | -1.05±0.70 | -0.72±0.67 | -1.29 |  |  |  |
|  |  | T2 | -1.12±0.75 | -0.80±0.57 | -1.28 |  |  |  |
|  |  | Multiple comparison | - | - |  |  |  |  |
|  | Switch | T0 | -0.88±0.87 | -1.02±0.48 | 0.56 | 2.93 | 0.10 | 0.71 |
|  |  | T1 | -1.14±0.80 | -0.85±0.61 | -1.10 |  |  |  |
|  |  | T2 | -1.22±0.74 | -0.78±0.82 | -1.53 |  |  |  |
|  |  | Multiple comparison | - | - |  |  |  |  |
| Region | Repeat | T0 | -1.02±0.79 | -0.97±0.63 | -0.20 | 1.76 | 1.48 | 1.77 |
|  |  | T1 | -1.35±0.71 | -1.00±0.73 | -1.34 |  |  |  |
|  |  | T2 | -1.43±0.86 | -0.92±0.57 | -1.84 |  |  |  |
|  |  | Multiple comparison | T0>T1, T0>T2 | - |  |  |  |  |
|  | Switch | T0 | -1.10±0.76 | -1.11±0.69 | 0.03 | 3.69^*^ | 0.69 | 1.59 |
|  |  | T1 | -1.38±0.80 | -1.13±0.82 | -0.83 |  |  |  |
|  |  | T2 | -1.60±0.94 | -0.88±0.83 | -2.21^*^ |  |  |  |
|  |  | Multiple comparison | T0>T2^#^ | - | - |  |  |  |

Notes: ^*^: *P*<0.05; ^#^: *P*<0.05 and *P*_adj>0.05
